# Challenges in artificial intelligence for polyp detection

**DOI:** 10.1111/den.14279

**Published:** 2022-03-22

**Authors:** Yuichi Mori, Masashi Misawa, Shin‐ei Kudo

**Affiliations:** ^1^ 6305 Clinical Effectiveness Research Group Institute of Health and Society University of Oslo Oslo Norway; ^2^ Section for Gastroenterology Department of Transplantation Medicine Oslo University Hospital Oslo Norway; ^3^ Digestive Disease Center Showa University Northern Yokohama Hospital Kanagawa Japan

Picking up advanced lesions with a subtle appearance has been a big challenge during colonoscopy. Obviously, type 0−IIc neoplasms in the Paris classification^1^ and laterally spreading tumors without granules (so called LST‐NG) including sessile serrated lesions are the typical examples of these complicated lesions.^2^ Although they are easy to be overlooked, the rate of including high‐grade dysplasia and cancer in these lesions is much higher than other types of colorectal neoplasia such as protruded/pedunculated polyps. They, therefore, may have something to do with the cause of postcolonoscopy colorectal cancer.^3^ Thus, eliminating overlooking of these kinds of lesions is clinically relevant. However, it is not that easy to change the current situation. An interesting study relevant to this issue was published in 2020 in Japan. According to this randomized controlled trial in which 2166 patients were followed up 3 years after polypectomy, more than 50% of the detected advanced neoplasia (15/29) in the follow‐up colonoscopy were actually LST‐NG.^3^ Furthermore, in this trial the baseline colonoscopies with polypectomies were offered twice by experienced endoscopists. Nevertheless, significant numbers of LST‐NG developed. Subtle and advanced lesions are still a big challenge, even when we have a chance to undergo high‐quality colonoscopy screening.

In this issue of *Digestive Endoscopy*, Ahmad *et al*. tackled this important clinical challenge with the aid of an artificial intelligence (AI) tool that is designed to detect polyps during colonoscopy.^4^ As is well known, AI for colonoscopy is leading the academic field of AI‐medicine in terms of the number of randomized controlled trials that showed the benefits of detecting more numbers of adenomas. However, the latest meta‐analysis revealed that these benefits were mainly attributed to the increased detection of nonadvanced adenoma, and thus whether AI can increase detection of subtle and advanced lesions such as LST‐NG is debated.^5^ Ahmad *et al*.^4^ intentionally picked up this important clinical question and clarified the role of AI in detecting these challenging lesions. According to the study results, their AI tool was able to detect 80% of the subtle lesions, while experienced and less‐experienced endoscopists detected only 37% and 11% of them, respectively. What is distinct in their study design is that they used prospectively produced, video‐based test data that only included image frames in which subtle and advanced lesions appeared in the periphery or distance of the visual field. In addition, to further understand the power of the AI tools in a more challenging situation, they analyzed the first few seconds of the appearance of these polyps. Surprisingly, AI outperformed endoscopists’ performance even under this challenging setting.

We think this amazing performance of AI could be generalizable. Recently, similar effectiveness against elusive polyps was also shown by another research team led by Google Health,^6^ in which the developed AI tool identified 85% of polyps in the field of view for less than 2 s. Therefore, we may expect this strength of AI for polyp detection, regardless of minor differences among different AI algorithms. This hypothesis is also supported by actual clinical trials. Recently published tandem randomized controlled trial in Japan showed that two LST‐NG‐type advanced adenomas (13.3% of all the advanced adenomas) were missed with standard colonoscopy but immediately picked up in the following AI‐assisted colonoscopy.^7^ It would be interesting to see how more accumulated data in randomized controlled trials and meta‐analyses will change our mind towards the use of AI in terms of detection of subtle, elusive, but advanced lesions. Actually, another meta‐analysis indicated that the use of AI increased the mean number of advanced adenomas per colonoscopy.^8^ We lack confidence in this benefit probably due to the lack of sample size in each single clinical trial. At the same time, researchers in the AI field should emphasize that the use of AI covers only recognition error of endoscopists, but not exposure error during endoscopy. Fundamental training to maximize mucosal exposure should always be valued.

Approximately 5 years have passed since the explosion of AI in medicine in the research field.^9^ We now know that the use of AI is a kind of fashion, and thus we should differentiate what is overestimated and what is appropriately evaluated based on scientific evidence. We guess AI for polyp detection may be the exception to the overestimated trends in AI‐medicine, which is strongly supported by both robustly designed randomized controlled trials and high‐quality benchmark tests such as the study published in this issue of *Digestive Endoscopy*.^4^ Ultimately, future studies in the field of AI in colonoscopy need to be focused on more robust outcome measures such as cancer prevention effect.^10^ The key (and the most challenging) question is “Does AI saves lives?”

## Conflict of Interest

Authors Y.M. and M.M. have received consulting fees and honoraria for lectures from Olympus, and ownership interest from Cybernet System Corp. M.M. is an Associate Editor of *Digestive Endoscopy*. The other author declares no conflict of interest for this article.

## Funding Information

None.
